# Prevalence and Genetic Characterization of* Cryptosporidium* Infection in Java Sparrows (*Lonchura oryzivora*) in Northern China

**DOI:** 10.1155/2017/2318476

**Published:** 2017-07-04

**Authors:** Qiu-Xia Yao, Xiao-Xuan Zhang, Kai Chen, Jian-Gang Ma, Wen-Bin Zheng, Xiao-Qin Xu, Xing-Quan Zhu

**Affiliations:** ^1^State Key Laboratory of Veterinary Etiological Biology, Key Laboratory of Veterinary Parasitology of Gansu Province, Lanzhou Veterinary Research Institute, Chinese Academy of Agricultural Sciences, Lanzhou, Gansu Province 730046, China; ^2^Jiangsu Co-Innovation Center for Prevention and Control of Important Animal Infectious Diseases and Zoonoses, Yangzhou University College of Veterinary Medicine, Yangzhou, Jiangsu Province 225009, China; ^3^College of Animal Science and Technology, Jilin Agricultural University, Changchun, Jilin Province 130118, China

## Abstract

Cryptosporidiosis is a cosmopolitan parasitosis that affects a wide range of hosts including birds. As information concerning* Cryptosporidium* in birds is limited, the present study examined the prevalence and genotypes of* Cryptosporidium* in Java sparrows in Beijing and Shangqiu, northern China. Three hundred and fifty fecal samples were collected from Java sparrows (*Lonchura oryzivora*, 225 white Java sparrows and 125 gray Java sparrows) in Beijing and Shangqiu in October 2015, and the samples were examined by PCR amplification of the small subunit ribosomal RNA (SSU rRNA) gene. The overall* Cryptosporidium* prevalence is 13.42% (47/350), with 16.44% (37/225) in white Java sparrows and 8.00% (10/125) in gray Java sparrows.* Cryptosporidium* prevalence was 9.82% (16/163) in Java sparrows from Beijing and 16.58% (31/187) in Java sparrows from Shangqiu. The prevalence of* Cryptosporidium* in females and males was 40.63% (26/64) and 7.34% (21/286), respectively. The* Cryptosporidium* prevalence in Java sparrows of different ages varied from 10.47% to 16.33%. Sequence analysis of the SSU rRNA gene revealed that all the samples represented* C. baileyi*. This is the first report of* Cryptosporidium* in gray Java sparrows in China, which extend the host range for* C. baileyi*. These results provide baseline information for further studies of molecular epidemiology and control of* Cryptosporidium* infection in poultry in China.

## 1. Introduction

Enteric parasite* Cryptosporidium* is one of the most important causative agents of gastrointestinal illnesses in humans and animals [1–3]. Food and water contaminated by* Cryptosporidium* oocysts are the most important resources for human infection [3–5]. Moreover, it can be transmitted to humans by direct contact with infected animals [3, 6]. Human infection with* Cryptosporidium* may show symptoms of diarrheal illness and some other severe diseases.* Cryptosporidium* infection in birds may present symptoms of respiratory disease, digestive symptoms, and renal disease [7, 8].

*Cryptosporidium* infection in birds was firstly recorded in 1929. So far, over 30 avian species have been reported as the hosts of* Cryptosporidium* in the world [7]. Respiratory symptoms frequently occur in birds, and this infection leads to higher morbidity and mortality in birds, especially in broiler chickens [7]. Recently, more than 17* Cryptosporidium* species/genotypes (*C. galli*,* C. baileyi*,* C. meleagridis*,* C. hominis*,* C. parvum*,* C. muris*,* Cryptosporidium* avian I–V, goose genotypes I–IV, duck genotype, and Eurasian woodcock genotype) have been recorded in birds worldwide [7, 9–13]. Of these,* C. hominis*,* C. parvum*, and* C. meleagridis* are well-known causative agents of human infection [12].

In view of such severe situations, a number of studies concerning* Cryptosporidium* prevalence and genotypes in birds have been conducted worldwide. In China, a few studies on* Cryptosporidium* infection in birds have been reported in Henan [12], Qinghai [14], and Zhejiang [15] provinces. A preliminary survey indicated that* Cryptosporidium* could infect white Java sparrows (*Lonchura oryzivora*) [12], but the number of sampled birds is too small (*n* = 25), which may not reflect the actual prevalence of* Cryptosporidium* in this bird. Also, no information is available about the prevalence of* Cryptosporidium* in gray Java sparrows in China. Therefore, we investigated the* Cryptosporidium* prevalence in Java sparrows (*Lonchura oryzivora*) in China and assessed its associated risk factors.

## 2. Materials and Methods

### 2.1. The Investigated Sites

The survey was conducted in Beijing and Shangqiu cities (two main locations of birds' production), northern China. Beijing city (39°26′–41°03′N, 115°25′–117°30′E) lies to the south of the Yanshan Mountains with an average altitude of 43.5 m, annual precipitation of 626 mm, and average annual temperature of 12.6°C. Shangqiu city (114°82′–116°45′E, 33°98′–34°80′N) is located in Henan province and has a subhumid warm temperate continental monsoonal climate. The altitude of Shangqiu city ranges from 30 m to 70 m, and the average annual temperature is 14.2°C.

### 2.2. Collection and Preparation of Samples

A total of 350 fecal samples were collected randomly from 225 white Java sparrows (*Lonchura oryzivora*) and 125 gray Java sparrows (*Lonchura oryzivora*), of which 163 were obtained from Beijing and 187 were obtained from Shangqiu from pet shops in 2015. Fecal samples were collected by using aseptic cotton and then filtered (0.3 mm wire mesh), and the filtrate was transferred into a 1.5 mL tube. All the samples were stored at 4°C until used for further analysis. Genomic DNA was extracted from feces using a Stool DNA kit (OMEGA, USA) according to the manufacturer's instructions. The DNA samples were stored at −20°C until used. Information about species, origin, gender, and age of Java sparrows was obtained and listed in Table 1.

### 2.3. PCR Amplification

*Cryptosporidium* species/genotypes were determined by nested PCR amplification of the small subunit ribosomal RNA (SSU rRNA) gene using primers F1 (5′-CCCATTTCCTTCGAAACAGGA-3′) and R1 (5′-TTCTAGAGCTAATACATGCG-3′) for primary amplification and F2 (5′-AAGGAGTAAGGAACAACCTCCA-3′) and R2 (5′-GGAAGGGTTGTATTTATTAGATAAAG-3′) for secondary amplification [16, 17]. PCR reaction (25 *μ*L) was composed of 1x PCR buffer (Mg^2+^-free), 2 mM MgCl_2_, 200 *μ*M of each deoxyribonucleoside triphosphate (dNTP), 0.4 *μ*M of each primer, 0.2 U of HotStart* Taq* DNA polymerase (Takara, Dalian, China), and 2 *μ*L of DNA template. The cycling conditions were 5 min at 95°C for initial denaturation and then 35 cycles of 45 s at 94°C, 45 s at 55°C, and 1 min at 72°C, followed by final extension at 72°C for 10 min. Both positive and negative controls were included in each test. PCR products were observed under UV light after electrophoresis in 1.5% agarose gel containing GoldView™ (Solarbio, Beijing, China).

### 2.4. Sequencing Analyses

The positive PCR products of Java sparrows were sequenced by GenScript Company (Nanjing, China).* Cryptosporidium* species were determined by alignment with known reference sequences available in GenBank using BLAST (https://www.ncbi.nlm.nih.gov/BLAST/). Representative nucleotide sequences were deposited in GenBank under accession numbers KY034418-KY034427 and KY962159.

### 2.5. Statistical Analysis

The variation in* Cryptosporidium* prevalence (*y*) of Java sparrows of different geographical location (*x*1), age (*x*2), species (*x*3), and gender (*x*4) was analyzed by *χ*^2^ test using SPSS 19.0. In the multivariable regression analysis, each of these variables was included in the binary logit model as an independent variable. The best model was judged by Fisher's scoring algorithm. All tests were two-sided, and values of probability (*P*) < 0.05 were considered statistically significant. Odds ratios (ORs) and their 95% confidence intervals (95% CIs) were estimated to explore the strength of the association between* Cryptosporidium* positivity and the conditions tested.

## 3. Results and Discussion

Forty-seven out of 350 Java sparrows (13.42%) were tested to be* Cryptosporidium*-positive by nested PCR amplification of the SSU rRNA gene (Table 1).* Cryptosporidium* prevalence was 9.82% (16/163) in Beijing and 16.58% (31/187) in Shangqiu (Table 1). The prevalence in white Java sparrows and gray Java sparrows was 16.44% (37/225) and 8.00% (10/125), respectively (Table 1). Moreover, female Java sparrows (40.63%, 26/64) had a statistically higher* Cryptosporidium* prevalence than males (7.34%, 21/286) (Table 1). The infection rates of* Cryptosporidium* in the different age ranged from 10.47% to 16.33%, with the highest prevalence in the 6–8-month group (Table 1), but the difference was not statistically significant among age groups (*P* > 0.05). Species and sex of Java sparrows were considered as main risk factors to influence the positive rate significantly.

Analyses of the obtained SSU rRNA sequences indicated that all of the 47 isolates represented* C. baileyi*. These sequences were deposited in GenBank under accession numbers KY034418-KY034427 and KY962159. Comparative analyses of all the obtained SSU rRNA sequences with that of the two known reference sequences (*C. baileyi,* GU816040 and* C. baileyi*, AF262324) indicated that no variation was found among them.

In this study, the prevalence of* Cryptosporidium* in Java sparrows is 13.42%, which is higher than the 4.86% prevalence in birds in Brazil by PCR [11], 8.1% in white Java sparrows in China by Sheather's sugar flotation technique [12], and 3.22% in parrots in China by ELISA [2]. Moreover, the* Cryptosporidium* prevalence of the present study was lower than that in pigeons in Thailand (25%) [18]. Different age distributions, test methods, geological environment conditions, and the breeding density may contribute to the difference of* Cryptosporidium* prevalence [2, 19].

In the present study, all of the obtained 47* Cryptosporidium* isolates belong to* C. baileyi*.* C*.* baileyi* is approximately the most common avian* Cryptosporidium* [20, 21] because it has been reported in a wide range of avian hosts including chickens, turkeys, ducks, black-billed magpie, common myna, crested lark, Gouldian finch, red-billed leiothrix, mixed-bred falcons, zebra finch, rose-ringed parakeet, white Java sparrow, grey partridge, saffron finch, and ruddy shelducks [7, 12, 20–22].

## 4. Conclusion

The present study revealed overall* Cryptosporidium* prevalence of 13.42% (47/350) with 16.44% (37/225) in white Java sparrows and 8.00% (10/125) in gray Java sparrows. This is the first report of* C. baileyi* in gray Java sparrows in China, which extend the host for* C. baileyi*. These results provide baseline information for further studies of molecular epidemiology and control of* Cryptosporidium* infection in poultry in China.

## Figures and Tables

**Table 1:** Factors associated with prevalence of *Cryptosporidium* in Java sparrows in Beijing and Shangqiu, northern China.

Factor	Category	Number positive	Number tested	Prevalence (%) (95% CI)	OR (95% CI)	*P* value
Region	Beijing	16	163	9.82 (5.26–14.38)	Reference	0.06
	Shangqiu	31	187	16.58 (11.25–21.91)	0.55 (0.29–1.04)	

Species	White Java sparrow	37	225	16.44 (11.60–21.28)	Reference	0.03
	Gray Java sparrow	10	125	8.00 (3.24–12.76)	2.26 (1.08–4.73)	

Sex	Female	26	64	40.63 (28.60–52.66)	Reference	<0.01
	Male	21	286	7.34 (2.22–12.46)	8.63 (4.43–16.84)	

Age	3~5 months	11	92	11.95 (5.33–18.57)	Reference	0.38
	6~8 months	25	153	16.33 (10.47–22.19)	0.70 (0.49–2.82)	
	>8 months	11	105	10.47 (4.61–16.33)		

*Total*		47	350	13.42 (9.85–16.99)		

## References

[1] Li Q., Li L., Tao W. (2016). Molecular investigation of *Cryptosporidium* in small caged pets in northeast China: host specificity and zoonotic implications. *Parasitology Research*.

[2] Zhang X.-X., Zhang N.-Z., Zhao G.-H., Zhao Q., Zhu X.-Q. (2015). Prevalence and genotyping of *Cryptosporidium* infection in pet parrots in North China. *BioMed Research International*.

[3] Zhao G.-H., Du S.-Z., Wang H.-B. (2015). First report of zoonotic *Cryptosporidium* spp., Giardia intestinalis and Enterocytozoon bieneusi in golden takins (*Budorcas taxicolor bedfordi*). *Infection, Genetics and Evolution*.

[4] Feng Y., Xiao L. (2011). Zoonotic potential and molecular epidemiology of *Giardia* species and giardiasis. *Clinical Microbiology Reviews*.

[5] Xiao L. (2010). Molecular epidemiology of cryptosporidiosis: an update. *Experimental Parasitology*.

[6] Šlapeta J. (2013). Cryptosporidiosis and *Cryptosporidium* species in animals and humans: a thirty colour rainbow?. *International Journal for Parasitology*.

[7] Ryan U. (2010). *Cryptosporidium* in birds, fish and amphibians. *Experimental Parasitology*.

[8] Xiao L., Fayer R., Ryan U., Upton S. J. (2004). *Cryptosporidium* Taxonomy: recent advances and implications for public health. *Clinical Microbiology Reviews*.

[9] Gomes R. S., Huber F., Da Silva S., Do Bomfim T. C. B. (2012). *Cryptosporidium* spp. parasitize exotic birds that are commercialized in markets, commercial aviaries, and pet shops. *Parasitology Research*.

[10] Nakamura A. A., Meireles M. V. (2015). *Cryptosporidium* infections in birds - a review. *Revista Brasileira de Parasitologia Veterinaria*.

[11] Nakamura A. A., Simões D. C., Antunes R. G., da Silva D. C., Meireles M. V. (2009). Molecular characterization of *Cryptosporidium* spp. from fecal samples of birds kept in captivity in Brazil. *Veterinary Parasitology*.

[12] Qi M., Wang R., Ning C. (2011). *Cryptosporidium* spp. in pet birds: genetic diversity and potential public health significance. *Experimental Parasitology*.

[13] Ryan U., Xiao L., Read C., Zhou L., Lal A. A., Pavlasek I. (2003). Identification of novel *Cryptosporidium* genotypes from the Czech Republic. *Applied and Environmental Microbiology*.

[14] Wang R., Wang F., Zhao J. (2012). *Cryptosporidium* spp. in quails (*Coturnix coturnix japonica*) in Henan, China: molecular characterization and public health significance. *Veterinary Parasitology*.

[15] Wang L., Xue X., Li J., Zhou Q., Yu Y., Du A. (2014). Cryptosporidiosis in broiler chickens in Zhejiang Province, China: molecular characterization of oocysts detected in fecal samples. *Parasite*.

[16] Qin S. Y., Zhang X. X., Zhao G. H. (2014). First report of *Cryptosporidium* spp. in white yaks in China. *Parasites Vectors*.

[17] Zhang X.-X., Cong W., Ma J.-G. (2016). First report of *Cryptosporidium* canis in farmed Arctic foxes (*Vulpes lagopus*) in China. *Parasites and Vectors*.

[18] Koompapong K., Mori H., Thammasonthijarern N. (2014). Molecular identification of *Cryptosporidium* spp. in seagulls, pigeons, dogs, and cats in Thailand. *Parasite*.

[19] Huang J., Yue D., Qi M. (2014). Prevalence and molecular characterization of *Cryptosporidium* spp. and *Giardia duodenalis* in dairy cattle in Ningxia, northwestern China. *BMC Veterinary Research*.

[20] Huber F., da Silva S., Bomfim T. C. B., Teixeira K. R. S., Bello A. R. (2007). Genotypic characterization and phylogenetic analysis of *Cryptosporidium* sp. from domestic animals in Brazil. *Veterinary Parasitology*.

[21] Wang R., Jian F., Sun Y. (2010). Large-scale survey of *Cryptosporidium* spp. in chickens and Pekin ducks (*Anas platyrhynchos*) in Henan, China: prevalence and molecular characterization. *Avian Pathology*.

[22] Van Zeeland Y. R. A., Schoemaker N. J., Kik M. J. L., Van Der Giessen J. W. B. (2008). Upper respiratory tract infection caused by *Cryptosporidium baileyi* in three mixed-bred falcons (*Falco rusticolus* x *Falco cherrug*). *Avian Diseases*.

